# Dynamic Modeling and Analysis of Channel Characterization for RIS-Aided V2V Wireless Communication Systems

**DOI:** 10.3390/s26041177

**Published:** 2026-02-11

**Authors:** Lin Guo, Jiahao Ge, Biyang Tu, Chao Meng, Xuejun Zhang

**Affiliations:** 1 School of Modern Posts, Nanjing University of Posts and Telecommunications, Nanjing 210003, China; guolin@njupt.edu.cn (L.G.);; 2 College of Electronic and Optical Engineering and College of Flexible Electronics (Future Technology), Nanjing University of Posts and Telecommunications, Nanjing 210003, China

**Keywords:** RIS, UAV communications, end-to-end channel model, propagation characteristics

## Abstract

Recently, wireless channel in vehicle-to-vehicle (V2V) communications is highly challenging due to rapid signal fluctuations, multi-path fading, and frequent obstructions. To mitigate these issues, this paper proposes a three-dimensional (3D) end-to-end channel model for reconfigurable intelligent surface (RIS)-assisted V2V wireless channels. The proposed channel model incorporates the motions of the mobile transmitter (MT), mobile receiver (MR), and a UAV-mounted RIS, capturing time-varying propagation characteristics. Key contributions include the derivation of exact analytical expressions for the level crossing rate (LCR) of the Rician fading envelope, along with efficient deterministic and stochastic simulation methods. Numerical results analyze the impact of RIS motion direction and scatterer distribution on the LCR, providing theoretical insights for optimizing RIS-assisted V2V systems in wireless communication environments.

## 1. Introduction

The rapid advancement of intelligent transportation systems imposes unprecedented demands on wireless communication networks [1]. Vehicle-to-vehicle (V2V) communication plays a crucial role in enabling smart connectivity, supporting applications that require ultra-reliable low-latency transmission alongside high data throughput [2]. In the existing literature, channel models are broadly categorized into deterministic and stochastic approaches. Deterministic models, exemplified by ray-tracing techniques, typically require extensive field measurements to characterize radio wave propagation paths and field strength distributions. While such models achieve high accuracy in representing specific propagation environments, their substantial computational cost and time-consuming nature restrict scalability, particularly in emerging systems such as RIS-assisted V2V communications. In contrast, stochastic models are constructed by characterizing channel fading as a random process, thereby offering greater flexibility and generalizability. Through appropriate parameterization, these models can be adapted to a wide range of communication scenarios, facilitating broader applicability in system design and performance evaluation. However, V2V wireless channels present significant challenges due to rapid signal fluctuations, severe multi-path fading, and frequent blockages caused by vehicle mobility and surrounding infrastructure such as trees and buildings [3]. The line-of-sight (LoS) link, essential for stable and high-fidelity communication, can be easily obstructed, leading to severe degradation in performance and potentially jeopardizing critical operations. To address these challenges, conventional approaches often rely on increased transmission power or additional infrastructure deployment, which are either energy-inefficient or cost-prohibitive. Recent studies have explored artificial intelligence (AI)-based solutions to enhance communication quality, while others have investigated security frameworks for wireless networks or analyzed the prospects of 6G in various service domains [4]. In parallel, reconfigurable intelligent surfaces (RISs) have emerged as a transformative technology capable of proactively shaping the wireless propagation environment. Composed of arrays of low-cost, passive elements, each capable of independently adjusting the phase and amplitude of incident electromagnetic waves, RISs can constructively combine signals at the intended receiver [5,6,7]. The authors in [8] proposed using RISs as an alternative to backscatter devices for achieving symbol-level reflection on the backscatter link, leading to a novel RIS-aided cell-free massive MIMO symbiotic radio system, which overcomes the conventional approach’s limitation of low spectral efficiency on the backscatter link. This effectively establishes a virtual LoS link even when the physical path is obstructed, thereby enhancing signal strength, extending coverage, and improving spectral efficiency.

Furthermore, the integration of RISs into vehicular networks, particularly in V2V communications, introduces a promising yet complex paradigm [9,10]. Unlike static configurations, the mobility of both transceivers and potentially the RIS itself-for instance, when mounted on an unmanned aerial vehicle-imparts distinct time-varying characteristics to the channel. Propagation distances, angles of departure (AoDs), and angles of arrival (AoAs) become explicit functions of time, resulting in inherent non-stationarity where statistical properties evolve dynamically [11]. Accurate modeling of this temporal variability is not merely an academic pursuit but a fundamental prerequisite for developing effective signal processing algorithms, resource allocation strategies, and performance evaluation frameworks for RIS-assisted V2V systems [12]. Thus far, there has been a substantial body of research on RIS-assisted V2V wireless channel modeling. As shown in Table 1, in [13], the authors proposed a RIS-assisted V2V channel model by generating a geometry-based channel via discrete Fourier transform beamforming, thereby addressing the issue of high computational complexity. In [14], the authors conducted a comprehensive comparative performance evaluation of ray-tracing and geometry-based channel modeling techniques for RIS-assisted V2V communications at mmWave frequencies. Another study in [15] considered a RIS-aided V2V network model using power-domain NOMA and derived closed-form expressions for outage probability, ergodic capacity, and energy efficiency to evaluate system performance. In [16], the authors examined the impact of environmental factors such as rain and fog, as well as vehicle mobility, on the channel characteristics of V2V communication systems. Furthermore, in [17], the performance of physical layer security was investigated in a V2V system where a transmitter vehicle employs dual RISs to transmit confidential information to legitimate receivers under a non-orthogonal multiple access scheme in the presence of an eavesdropping vehicle. Although existing research has advanced the modeling of RIS-assisted channels, notable gaps remain, especially in the V2V context. Many studies rely on the static assumptions, which fail to capture the temporal non-stationarity characteristic of high-mobility vehicular scenarios. Moreover, there is limited work examining second-order statistical properties for RIS-enhanced V2V channels, such as the level crossing rate (LCR) and average fade duration (AFD). These metrics are critical for characterizing fading dynamics, estimating packet error rates, and designing robust interleaving and diversity schemes [18,19,20]. To bridge these gaps, this paper develops a three-dimensional (3D) end-to-end time-varying channel model for RIS-assisted V2V communications, accounting for the motions of the mobile transmitter, mobile receiver, and a UAV-mounted RIS. The model captures the non-stationary propagation characteristics induced by mobility and provides exact analytical expressions for the LCR of the Rician fading envelope. Additionally, efficient deterministic and stochastic simulation methods are introduced. Numerical analyses illustrate the influence of RIS movement direction and scatterer distribution on the LCR, offering theoretical insights for the optimization of RIS-assisted V2V systems in dynamic wireless environments. The key contributions of this work are summarized as follows:

- We develop a 3D end-to-end channel model for an RIS-assisted V2V communication system, considering the motions of the MT, MR, and the RIS-mounted UAV. The model incorporates both the controllable RIS reflection link and the scattered NLoS link from clusters, providing a holistic view of the propagation environment.
- The model explicitly derives the time-varying expressions for crucial parameters, including propagation distances, AoDs, AoAs, and complex impulse responses for both the RIS and cluster links, capturing the inherent temporal non-stationarity.
- We derive exact analytical expressions for the key second-order statistic, the LCR of the envelope for the proposed Rician fading channel model. This derivation meticulously accounts for the contributions from both the RIS link and the NLoS scattering link through their respective spectral moments.
- We provide efficient deterministic and stochastic simulation methods for generating the discrete angles needed to realize the channel model and compute the simulated LCR, ensuring computational practicality.
- Through extensive numerical analysis and simulations, we investigate the impact of various system parameters, such as the motion direction of the RIS and the distribution of scatterers on the LCR. We offer theoretical insights explaining how aligning the RIS motion with dominant propagation paths affects the Doppler spread and, consequently, the fading dynamics.

The rest of this paper is organized as follows. Section 2 deduces the complex CIRs for various propagation links in the proposed channel model. In Section 3, the propagation properties of the proposed channel model are deduced and examined. In Section 4 and Section 5, we separately present the simulation findings and some related discussion. Finally, some conclusions are drawn in Section 6.

Notations: The boldface uppercase, boldface lowercase, and lowercase letters such as X, x, and *x* represent the matrix, vector, and scalar, respectively. E[·] represents the expectation operation, ${\left[\;\cdot \;\right]}^{T}$ represents the transpose operation, and ${\left(\cdot \right)}^{*}$ represents the complex conjugate operation. ∥·∥ is the Frobenius norm, and $j=\sqrt{-1}$ denotes the imaginary unit.

## 2. System Channel Model Description

### 2.1. Geometry of the Proposed Channel Model

**Figure 1:**
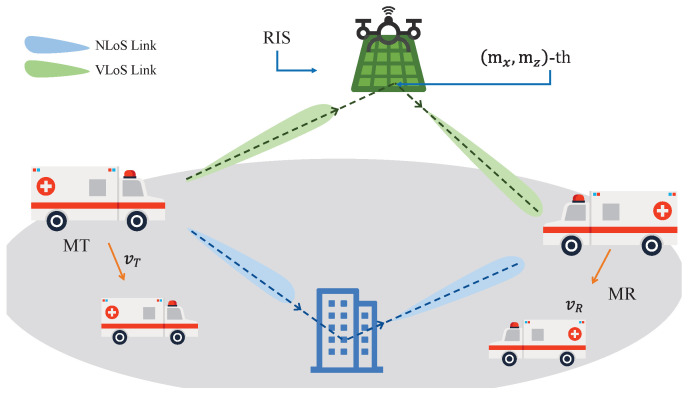
Propagation distances and angles in the proposed RIS-assisted V2V channel model for different links.

**Figure 2:**
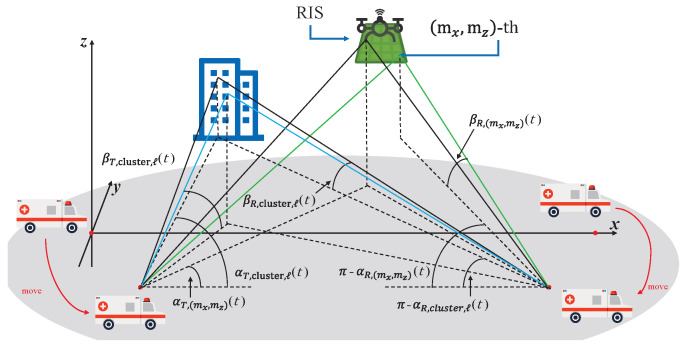
Schematic diagram of some parameters of the proposed channel model.

Consider a RIS-assisted channel model for V2V propagation environment as shown in Figure 1. The transmitter (MT) and receiver (MR) move at speeds $v_{T}$ and $v_{R}$, respectively [21]. The RIS consists of $M_{x}\times M_{z}$ units and is mounted on a UAV. In addition, the dimensions of each unit in RIS are denoted as $d_{M_{x}}$ and $d_{M_{z}}$. For simplicity, the UAV and RIS are treated as a single entity and are assumed to move at a uniform velocity $v_{\mathrm{RIS}}$. In the proposed RIS-assisted V2V MIMO channel model, the movements of the MT and MR will cause time-varying model parameters, including propagation distances and angles, resulting in the temporal non-stationary property of the channel. In light of this, time-varying model parameters, including time-varying propagation distances and angles, are required to be derived to capture the non-stationary channel propagation characteristics in the time domain [22]. As shown in Figure 2, the wave emitted by the MT goes through two different propagation paths before arriving at the MR, which constitute the multi-path propagations of the proposed channel model. Namely, (i) the waves impinges on RIS before arriving at the MR, i.e., MT→RIS→MR link; (ii) the wave impinges on the cluster before arriving at the MR, i.e., MT→clusters→MR link.

In the initial motion stage of MT and MR, a global Cartesian coordinate system is created, where the MR is located at a distance $D_{0}$ from the MT, and the UAV maintains an altitude $H_{0}$ above the ground. To be specific, the line connecting the center of MT antenna array with the center of MR antenna array is defined as the *x*-axis, the *z*-axis is vertical and passes through the center of MT antenna array, and the *y*-axis is obtained in accordance with right-hand rule. It is worth noting that the suggested coordinate system is still maintained even when the MT and MR move from one position to another. As a result, the distance vectors from the origin of the global coordinate system to the centroids of the MT and MR antenna arrays can be respectively represented by [23].

(1) $$\begin{array}{c} d_{T}\left(t\right)={\left[\begin{array}{c} d_{T,x}\left(t\right),\;d_{T,y}\left(t\right),\;0 \end{array}\right]}^{T}, \end{array}$$

(2) $$\begin{array}{c} d_{R}\left(t\right)={\left[\begin{array}{c} d_{R,x}\left(t\right),\;d_{R,y}\left(t\right),\;0 \end{array}\right]}^{T}, \end{array}$$

where $d_{T,x}\left(t\right)=v_{T}t\cos\eta_{T}$, $d_{T,y}\left(t\right)=v_{T}t\sin\eta_{T}$, $d_{R,x}\left(t\right)=D_{0}+v_{R}t\cos\eta_{R}$, and $d_{R,y}\left(t\right)=v_{R}t\sin\eta_{R}$, in which $\eta_{T}$ is the angle between the motion direction of the MT and the *x*-axis, and $\eta_{R}$ is the angle between the motion direction of the MR and the *x*-axis. In addition, the distance vector from the origin of the global coordinate system to the cluster is denoted by $d_{\mathrm{cluster}}={\left[\;x_{\mathrm{cluster}},y_{\mathrm{cluster}},z_{\mathrm{cluster}}\;\right]}^{T}$, and the distance vectors from the origin of the global coordinate system to the center points of RIS is $d_{\mathrm{RIS}}\left(t\right)={\left[\;x_{\mathrm{RIS}}\left(t\right),y_{\mathrm{RIS}}\left(t\right),z_{\mathrm{RIS}}\left(t\right)\;\right]}^{T}$. In order to strengthen the generality of the RIS layout in the proposed channel model, $\theta_{\mathrm{RIS}}$ is introduced to describe the practical horizontal rotation angles of RIS. Then, the distance vectors from the origin of the global coordinate system to the $\left(m_{x},m_{z}\right)$-th ($m_{x}=1,2,\ldots M_{x};m_{z}=1,2,\ldots M_{z}$) unit in RIS expressed as 

(3) $$\;d_{m_{x},m_{z}}\left(t\right)=\;\;\left[\begin{array}{c} \;x_{m_{x},m_{z}}\;\\ y_{m_{x},m_{z}}\\ z_{m_{x},m_{z}} \end{array}\right]=\left[\begin{array}{c} \;x_{\mathrm{RIS}}\left(t\right)+\frac{1}{2}\left(2m_{x}-M_{x}-1\right)d_{M}\cos\theta_{\mathrm{RIS}}\;\\ y_{\mathrm{RIS}}\left(t\right)+\frac{1}{2}\left(2m_{x}-M_{x}-1\right)d_{M_{x}}\sin\theta_{\mathrm{RIS}}\\ z_{\mathrm{RIS}}\left(t\right)-\frac{1}{2}\left(2m_{z}-M_{z}-1\right)d_{M_{z}} \end{array}\right].$$

In the proposed channel model, the MT and MR are respectively composed of *P* and *Q* omnidirectional antennas in a uniform linear array (ULA). Distance vectors from the *p*-th (p=1,2,…,P) MT antenna and the *q*-th (q=1,2,…,Q) MR antenna to the origin of the global coordinate system can be respectively described as [24,25]

(4) $$\begin{array}{c} {\mathrm{Ant}}_{T}=\frac{P-2p+1}{2}\delta_{T}\;\left[\begin{array}{c} \;\cos\phi_{T}\cos\psi_{T}\;\\ \cos\phi_{T}\sin\psi_{T}\\ \sin\phi_{T} \end{array}\right]\;\;, \end{array}$$

(5) $$\begin{array}{c} {\mathrm{Ant}}_{R}=\frac{Q-2q+1}{2}\delta_{R}\;\left[\begin{array}{c} \;\cos\phi_{R}\cos\psi_{R}\;\\ \cos\phi_{R}\sin\psi_{R}\\ \sin\phi_{R} \end{array}\right]\;\;, \end{array}$$

Notably, the presented channel model utilizes uniform linear arrays (ULAs) at both the transmitter and receiver and restricts vehicle movement to a two-dimensional horizontal plane. While these simplifications enable analytical tractability, they can limit the model’s suitability for cases involving three-dimensional mobility or different array topologies, such as uniform planar arrays (UPAs). To extend the framework to UPAs or other geometries, the spatial-frequency representation may be expanded to incorporate azimuth and elevation dimensions, with corresponding modifications to the beamforming matrices. At the same time, the 2D mobility representation effectively captures the predominant horizontal motion characteristics. To accommodate 3D movement, the model can be enhanced by including time-varying velocity components along the vertical axis and updating the relevant geometric relationships and Doppler shift computations. These potential extensions highlight the adaptable nature of the proposed modeling approach. The $\delta_{T}$ and $\delta_{R}$ are the spacings between two neighboring MT/MR antennas, $\psi_{T}$ and $\phi_{T}$ are the azimuth and elevation angles of the MT antenna array, and $\psi_{R}$ and $\phi_{R}$ are the azimuth and elevation angles of the MR antenna array. Subsequently, the time-varying distances from the MT antenna and the MR antenna to the *ℓ*-th scatterer within the cluster can be respectively determined through taking the magnitudes of $\xi_{T,\mathrm{cluster},\ell }\left(t\right)=∥\;d_{T,\mathrm{cluster},\ell }\left(t\right)\;∥$ and $\xi_{R,\mathrm{cluster},\ell }\left(t\right)=∥\;d_{R,\mathrm{cluster},\ell }\left(t\right)∥$, i.e., 

(6) $$\begin{array}{ccc} d_{T,\mathrm{cluster},\ell }\left(t\right) & = & \left[\begin{array}{c} x_{\mathrm{cluster},\ell }-d_{T,x}\left(t\right)\\ y_{\mathrm{cluster},\ell }-d_{T,y}\left(t\right)\\ z_{\mathrm{cluster},\ell }-d_{T,z}\left(t\right) \end{array}\right]-{\mathrm{Ant}}_{T} \end{array}$$

(7) $$\begin{array}{ccc} d_{R,\mathrm{cluster},\ell }\left(t\right) & = & \left[\begin{array}{c} x_{\mathrm{cluster},\ell }-d_{R,x}\left(t\right)\\ y_{\mathrm{cluster},\ell }-d_{R,y}\left(t\right)\\ z_{\mathrm{cluster},\ell } \end{array}\right]-{\mathrm{Ant}}_{R} \end{array}$$

Moreover, the time-varying EAoD and AAoD of the wave from the MT to the *ℓ*-th scatterer within the cluster can be respectively represented as

(8) $$\begin{array}{c} \alpha_{T,\mathrm{cluster},\ell }\left(t\right)=\arctan\frac{y_{\mathrm{cluster},\ell }-d_{T,y}\left(t\right)}{x_{\mathrm{cluster},\ell }-d_{T,x}\left(t\right)}, \end{array}$$

(9) $$\beta_{T,\mathrm{cluster},\ell }\left(t\right)=\mathrm{arccot}\frac{\sqrt{{\left(x_{\mathrm{cluster},\ell }-d_{T,x}\left(t\right)\right)}^{2}+{\left(y_{\mathrm{cluster},\ell }-d_{T,y}\left(t\right)\right)}^{2}}}{z_{\mathrm{cluster},\ell }-d_{T,z}\left(t\right)}$$

In addition, the time-varying AAoA and EAoA of the wave from the *ℓ*-th scatterer to MR can be respectively described as

(10) $$\begin{array}{c} \alpha_{R,\mathrm{cluster},\ell }\left(t\right)=\arctan\frac{y_{\mathrm{cluster},\ell }-d_{R,y}\left(t\right)}{x_{\mathrm{cluster},\ell }-d_{R,x}\left(t\right)}, \end{array}$$

(11) $$\beta_{R,\mathrm{cluster},\ell }\left(t\right)=\mathrm{arccot}\frac{\sqrt{{\left(x_{\mathrm{cluster},\ell }-d_{R,x}\left(t\right)\right)}^{2}+{\left(y_{\mathrm{cluster},\ell }-d_{R,y}\left(t\right)\right)}^{2}}}{z_{\mathrm{cluster},\ell }}.$$

It is necessary to mention that the initial delay/angle/distance parameters can be identified once the geometric settings among MT, RIS, and MR are provided. The geometric correlations among MT, RIS, and MR can be used to obtain the values of model parameters after a time interval of *t* in accordance with the motion time/direction/velocity, as well as the values of values of initial parameters of MT and MR. Since we adopt the far-field assumption for RIS elements to investigate the proposed channel propagation characteristics, the RIS units share the same signal angles. In light of this, the AAoD and EAoD from the MT to the $\left(m_{x},m_{z}\right)$-th unit in RIS can be respectively represented as [26]

(12) $$\begin{array}{c} \alpha_{T,(m_{x},m_{z})}\left(t\right)=\arctan\frac{y_{m_{x},m_{z}}-d_{T,y}\left(t\right)}{x_{m_{x},m_{z}}-d_{T,x}\left(t\right)}, \end{array}$$

(13) $$\begin{array}{c} \beta_{T,(m_{x},m_{z})}\left(t\right)=\arctan\frac{z_{m_{x},m_{z}}-d_{T,z}\left(t\right)}{\sqrt{{\left(x_{m_{x},m_{z}}-d_{T,x}\left(t\right)\right)}^{2}+{\left(y_{m_{x},m_{z}}-d_{T,y}\left(t\right)\right)}^{2}}}. \end{array}$$

The AAoA and EAoA from the $\left(m_{x},m_{z}\right)$-th unit in RIS to the MR can be respectively expressed as

(14) $$\begin{array}{c} \alpha_{R,(m_{x},m_{z})}\left(t\right)=\arctan\frac{y_{m_{x},m_{z}}-d_{R,y}\left(t\right)}{x_{m_{x},m_{z}}-d_{R,x}\left(t\right)}, \end{array}$$

(15) $$\begin{array}{c} \beta_{R,(m_{x},m_{z})}\left(t\right)=\arctan\frac{z_{m_{x},m_{z}}}{\sqrt{{\left(x_{m_{x},m_{z}}-d_{R,x}\left(t\right)\right)}^{2}+{\left(y_{m_{x},m_{z}}-d_{R,y}\left(t\right)\right)}^{2}}}. \end{array}$$

In addition, the velocity of RIS can be described as

(16) $$\begin{array}{c} v_{\mathrm{RIS}}\left(t\right)={\left[\begin{array}{c} v_{\mathrm{RIS}}\left(t\right)\cos\gamma_{\mathrm{RIS}}\cos\eta_{\mathrm{RIS}},\;v_{\mathrm{RIS}}\left(t\right)\cos\gamma_{\mathrm{RIS}}\sin\eta_{\mathrm{RIS}},\;v_{\mathrm{RIS}}\left(t\right)\sin\eta_{\mathrm{RIS}} \end{array}\right]}^{T},\; \end{array}$$

where $\gamma_{\mathrm{RIS}}$ and $\eta_{\mathrm{RIS}}$ are the angles between the direction of RIS movement and the vertical and horizontal planes, respectively. As a result, the velocity of the MT relative to the RIS is given by

(17) $$\begin{array}{c} v_{T-\mathrm{RIS}}\left(t\right)={\left[\begin{array}{c} v_{T,x}\left(t\right)-v_{\mathrm{RIS},x}\left(t\right),\;v_{T,y}\left(t\right)-v_{\mathrm{RIS},y}\left(t\right),\;v_{T,z}\left(t\right)-v_{\mathrm{RIS},z}\left(t\right)\; \end{array}\right]}^{T},\; \end{array}$$

while the velocity of the MR relative to the RIS is similarly given by

(18) $$\begin{array}{c} v_{R-\mathrm{RIS}}\left(t\right)={\left[\begin{array}{c} v_{R,x}\left(t\right)-v_{\mathrm{RIS},x}\left(t\right),\;v_{R,y}\left(t\right)-v_{\mathrm{RIS},y}\left(t\right),\;v_{R,z}\left(t\right)-v_{\mathrm{RIS},z}\left(t\right)\; \end{array}\right]}^{T}.\; \end{array}$$

### 2.2. Complex CIRs of the Proposed Channel Model

The small-scale fading is caused by multi-path propagation components, which can be denoted by a Q×P complex matrix, i.e., $H_{\mathrm{TR}}^{\mathrm{small}}\left(t\right)={\left[h_{TR}\left(t,\tau \right)\right]}_{Q\times P}$. Here, $h_{TR}\left(t,\tau \right)$ stands for the complex CIR between the center point of the transmitting antenna array and that of the receiving antenna array in the proposed MIMO channel model, which can be described as [27]

(19) $$\begin{array}{c} \;h_{TR}\left(t,\tau \right)=\sqrt{\frac{\Omega_{TR}^{\mathrm{RIS}}\left(t\right)}{\Omega_{TR}^{\mathrm{RIS}}\left(t\right)+\Omega_{TR}^{\mathrm{NLoS}}\left(t\right)}}h_{TR}^{\mathrm{RIS}}\left(t\right)\delta \left(\tau -\tau^{\mathrm{RIS}}\left(t\right)\right)\\ +\sqrt{\frac{\Omega_{TR}^{\mathrm{NLoS}}\left(t\right)}{\Omega_{TR}^{\mathrm{RIS}}\left(t\right)+\Omega_{TR}^{\mathrm{NLoS}}\left(t\right)}}h_{TR}^{\mathrm{cluster}}\left(t\right)\delta \left(\tau -\tau^{\mathrm{cluster}}\left(t\right)\right), \end{array}$$

where $\tau^{\mathrm{RIS}}\left(t\right)=\left(\xi_{T,\mathrm{RIS}}\left(t\right)+\xi_{R,\mathrm{RIS}}\left(t\right)\right)/c$, with $\xi_{T,\mathrm{RIS}}\left(t\right)=∥d_{\mathrm{RIS}}\left(t\right)-d_{T}\left(t\right)∥$ and $\xi_{R,\mathrm{RIS}}\left(t\right)=∥d_{\mathrm{RIS}}\left(t\right)-d_{R}\left(t\right)∥$ respectively being the time-varying distances from the centers of MT and MR antenna arrays to that of RIS array, and *c* is the light speed; $\tau^{\mathrm{cluster}}\left(t\right)=\left(\xi_{T,\mathrm{cluster}}\left(t\right)+\xi_{R,\mathrm{cluster}}\left(t\right)\right)/c$, with $\xi_{T,\mathrm{cluster}}\left(t\right)=∥d_{\mathrm{cluster}}-d_{T}\left(t\right)∥$ and $\xi_{R,\mathrm{cluster}}\left(t\right)=∥d_{\mathrm{cluster}}-d_{R}\left(t\right)∥$ respectively being the time-varying distances from the centers of MT and MR antenna arrays to the center of the cluster in the real-time motion stage. The $\Omega_{TR}^{\mathrm{RIS}}\left(t\right)$ and $\Omega_{TR}^{\mathrm{NLoS}}\left(t\right)$ are the power gains of the propagation links through the RIS and cluster, respectively, which can be expressed as [28]

(20) $$\begin{array}{cc} \Omega_{TR}^{\mathrm{RIS}}\left(t\right)\; & =E\{\frac{\lambda^{2}dM_{x}dM_{z}}{{\left(4\pi \right)}^{3}}|\sum_{m_{x}=1}^{M_{x}}\sum_{m_{z}=1}^{M_{z}}\frac{\chi_{m_{x},m_{z}}\left(t\right)e^{j\varphi_{m_{x},m_{z}}\left(t\right)}}{\xi_{T,(m_{x},m_{z})}\left(t\right)\xi_{R,(m_{x},m_{z})}\left(t\right)}\\ \; & \;\times \;e^{-j\frac{2\pi }{\lambda }\left(\xi_{T,(m_{x},m_{z})}\left(t\right)+\xi_{R,(m_{x},m_{z})}\left(t\right)\right)}e^{j\frac{2\pi }{\lambda }v_{R}t\cos\left(\alpha_{R,(m_{x},m_{z})}\left(t\right)-\eta_{R}\right)\cos\beta_{R,(m_{x},m_{z})}\left(t\right)}\\ \; & \;\times \;e^{j\frac{2\pi }{\lambda }v_{T}t\cos\left(\alpha_{T,(m_{x},m_{z})}\left(t\right)-\eta_{T}\right)\cos\beta_{T,(m_{x},m_{z})}\left(t\right)\cos\gamma_{T}}e^{j\frac{2\pi }{\lambda }v_{T}t\sin\beta_{T,(m_{x},m_{z})}\left(t\right)\sin\gamma_{T}}{|}^{2}\}, \end{array}$$

(21) $$\begin{array}{c} \Omega_{\mathrm{TR}}^{\mathrm{cluster}}\left(t\right)=\frac{\lambda^{2}P_{\mathrm{cluster},\ell }\left(t\right)}{{\left(4\pi \right)}^{2}\;{\left(\xi_{T,\mathrm{cluster},\ell }\left(t\right)+\xi_{R,\mathrm{cluster},\ell }\left(t\right)\right)}^{2}}, \end{array}$$

where $P_{\mathrm{cluster},\ell }\left(t\right)=\exp\left(-\tau^{\mathrm{cluster}}\left(t\right)\frac{r_{\tau }-1}{r_{\tau }\sigma_{\tau }}\right)\cdot 10^{\frac{-Z_{\mathrm{cluster},\ell }}{10}}$ represents the cluster power. Here, $r_{\tau }$ and $\sigma_{\tau }$ represent the delay scale parameter and delay spread, respectively; $Z_{\mathrm{cluster},\ell }$ is a stochastic variable satisfying Gaussian distribution, i.e., $Z_{\mathrm{cluster},\ell }∼N\left(0,\zeta^{2}\right)$.

Furthermore, in Equation (19), $h_{TR}^{\mathrm{RIS}}\left(t\right)$ is the channel coefficient of the propagation links through the RIS, which can be expressed as

(22) $$\begin{array}{cc} h_{TR}^{\mathrm{RIS}}\left(t\right)\; & =\sqrt{\frac{1}{Y_{TR}^{\mathrm{RIS}}\left(t\right)}}\sum_{m_{x}=1}^{M_{x}}\sum_{m_{z}=1}^{M_{z}}\chi_{m_{x},m_{z}}\left(t\right)e^{j\varphi_{m_{x},m_{z}}\left(t\right)-j\frac{2\pi }{\lambda }\left(\xi_{T,(m_{x},m_{z})}\left(t\right)+\xi_{R,(m_{x},m_{z})}\left(t\right)\right)}\\ \; & \;\times \;e^{j\frac{2\pi }{\lambda }\left(v_{T-\mathrm{RIS}}\left(t\right)\cos<v_{T-\mathrm{RIS}},d_{T,m_{x},m_{z}}\left(t\right)>+v_{R-\mathrm{RIS}}\cos<d_{R,m_{x},m_{z}}\left(t\right),v_{R-\mathrm{RIS}}\left(t\right)>\right)t}, \end{array}$$

where λ is the wavelength; $\varphi_{m_{x},m_{z}}\left(t\right)$ and $\chi_{m_{x},m_{z}}\left(t\right)$ represent the reflection phase and amplitude of the $\left(m_{x},m_{z}\right)$-th unit in RIS, respectively; $Y_{TR}^{\mathrm{RIS}}\left(t\right)$ is the normalized factor of the MT→RIS→MR link to endow $h_{TR}^{\mathrm{RIS}}\left(t\right)$ with unit power; $\xi_{T,(m_{x},m_{z})}\left(t\right)$ and $\xi_{R,(m_{x},m_{z})}\left(t\right)$ respectively stand for the lengths from the MT and the MR antenna to the $\left(m_{x},m_{z}\right)$-th unit in RIS, i.e., $\xi_{T,(m_{x},m_{z})}\left(t\right)=∥d_{m_{x},m_{z}}-d_{T}\left(t\right)-{\mathrm{Ant}}_{T}∥$ and $\xi_{R,(m_{x},m_{z})}\left(t\right)=∥d_{m_{x},m_{z}}-d_{R}\left(t\right)-{\mathrm{Ant}}_{R}∥$. When RIS adopts the uniform reflection phase configuration, it is reasonable to assume that $\varphi_{m_{x},m_{z}}\left(t\right)$ follows the uniform distribution. However, when the phase regulation is discrete, $\varphi_{m_{x},m_{z}}\left(t\right)$ should be set to be some discrete values. $Y_{TR}^{\mathrm{RIS}}\left(t\right)$ can be expressed as [29]

(23) $$\begin{array}{cc} Y_{TR}^{\mathrm{RIS}}\left(t\right)\; & =E\{|\sum_{m_{x}=1}^{M_{x}}\sum_{m_{z}=1}^{M_{z}}\chi_{m_{x},m_{z}}\left(t\right)e^{j\varphi_{m_{x},m_{z}}\left(t\right)-j\frac{2\pi }{\lambda }\left(\xi_{T,(m_{x},m_{z})}\left(t\right)+\xi_{R,(m_{x},m_{z})}\left(t\right)\right)}\\ \; & \;\times \;e^{j\frac{2\pi }{\lambda }\left(v_{T-\mathrm{RIS}}\left(t\right)\cos<v_{T-\mathrm{RIS}}\left(t\right),d_{T,m_{x},m_{z}}\left(t\right)>+v_{R-\mathrm{RIS}}\left(t\right)\cos<d_{R,m_{x},m_{z}}\left(t\right),v_{R-\mathrm{RIS}}\left(t\right)>\right)t}{|}^{2}\}. \end{array}$$

For the NLoS propagation link via the cluster, the channel coefficient $h_{TR}^{\mathrm{cluster}}\left(t\right)$ can be described as 

(24) $$\begin{array}{ccc} h_{\mathrm{TR}}^{\mathrm{cluster}}\left(t\right)\; & = & \;\frac{1}{\sqrt{L}}\sum_{\ell =1}^{L}e^{j\varphi_{\ell }-j\frac{2\pi }{\lambda }\left(\xi_{T,\mathrm{cluster},\ell }\left(t\right)+\xi_{R,\mathrm{cluster},\ell }\left(t\right)\right)}\\ & & \;\times \;e^{j\frac{2\pi }{\lambda }\left(v_{T}\left(t\right)\cos<v_{T}\left(t\right),d_{T,\mathrm{cluster},\ell }\left(t\right)>+v_{R}\left(t\right)\cos<d_{R,\mathrm{cluster},\ell }\left(t\right),v_{R}\left(t\right)>\right)t}. \end{array}$$

It is worth mentioning that the proposed 3D channel model is inherently stochastic, allowing its framework to represent V2V communications in various environments via suitable parameter tuning. Such flexibility largely originates from modeling the random phase $\varphi_{\ell }$ of the complex fading envelope $h_{\mathrm{TR}}^{\mathrm{cluster}}\left(t\right)$ as uniformly distributed over [−π,π). As a result, the derived channel matrix H(t), which encapsulates the physical channel properties, maintains a stochastic nature. The initial delay/angle/distance parameters are derived from the randomly generated or measured activity. Scatterers in the same cluster are presumed to have roughly the same initial separation from the center of MR antenna array at different angles, i.e., $\xi_{R,\mathrm{cluster}}\left(0\right)\approx \xi_{R,\mathrm{cluster},1}\left(0\right)\cdots \approx \xi_{R,\mathrm{cluster},\ell }\left(0\right)\cdots \approx \xi_{R,\mathrm{cluster},L}\left(0\right)$.

## 3. LCR of V2V Rice Channels

The LCR at a threshold level *r*, L(r), quantifies how often the envelope crosses the level *r* in the positive/negative going direction. For Rician channels, it can be written as [30]

(25) $$\begin{array}{cc} \; & \;L\left(r\right)=\frac{2r\sqrt{K+1}}{\pi^{3/2}}\sqrt{\frac{b_{2}}{b_{0}}-\frac{b_{1}^{2}}{b_{0}^{2}}}e^{-K-\left(K+1\right)r^{2}}\\ \times \int_{0}^{\pi /2}\cosh\left(2\sqrt{K(K+1)}r\cos\theta \right)\left[e^{-{\left(\chi \sin\theta \right)}^{2}}+\sqrt{\pi }\chi \sin\theta \;\mathrm{erf}\left(\chi \sin\theta \right)\right]d\theta , \end{array}$$

where *K* is the Rician *K*-factor, cosh(·) is the hyperbolic cosine, erf(·) is the error function, and χ is given by

(26) $$\chi =\sqrt{\frac{Kb_{1}^{2}}{b_{0}b_{2}-b_{1}^{2}}}.$$

The key parameters (i.e., the spectral moments) $b_{m}$ (m=0,1,2) are given by

(27) $$b_{m}=b_{m}^{\mathrm{RIS}}+b_{m}^{\mathrm{NLoS}}.$$

For the RIS propagation link, the discrete angles $\alpha_{T,(m_{x},m_{z})}$, $\beta_{T,(m_{x},m_{z})}$, $\alpha_{R,(m_{x},m_{z})}$, and $\beta_{R,(m_{x},m_{z})}$ can be replaced by $\alpha_{T}$, $\beta_{T}$, $\alpha_{R}$, and $\beta_{R}$, respectively. In this case, the expression of the spectral moments can be expressed as

(28) $$\begin{array}{cc} b_{m}^{\mathrm{RIS}} & =\frac{\Omega_{\mathrm{TR}}^{\mathrm{RIS}}\left(t\right)}{2\Omega_{\mathrm{TR}}^{\mathrm{RIS}}\left(t\right)+2\Omega_{\mathrm{TR}}^{\mathrm{NLoS}}\left(t\right)}{\left(\frac{2\pi }{\lambda }\right)}^{m}\int_{-\pi /2}^{\pi /2}\int_{-\pi }^{\pi }f\left(\alpha_{T},\beta_{T}\right)\\ \; & \;\times [e^{j\varphi_{m_{x},m_{z}}\left(t\right)-j\frac{2\pi }{\lambda }\left(\xi_{T,(m_{x},m_{z})}\left(t\right)+\xi_{R,(m_{x},m_{z})}\left(t\right)\right)}\\ \; & \;\times \;e^{j\frac{2\pi }{\lambda }\left(v_{T\text{-}\mathrm{RIS}}\left(t\right)\cos\langle v_{T\text{-}\mathrm{RIS}}\left(t\right),d_{T,m_{x},m_{z}}\left(t\right)\rangle +v_{R\text{-}\mathrm{RIS}}\left(t\right)\cos\langle d_{R,m_{x},m_{z}}\left(t\right),v_{R\text{-}\mathrm{RIS}}\left(t\right)\rangle \right)t}{]}^{m}d\alpha_{T}d\beta_{T} \end{array}$$

For the NLoS propagation link, the discrete angles $\alpha_{T}^{\left(n\right)}$, $\beta_{T}^{\left(n\right)}$, $\alpha_{R}^{\left(n\right)}$, and $\beta_{R}^{\left(n\right)}$ can be replaced by $\alpha_{T}$, $\beta_{T}$, $\alpha_{R}$, and $\beta_{R}$, respectively. In this case, the expression of the spectral moments can be expressed as and for the NLoS path, the spectral moment can be specifically expressed as

(29) $$\begin{array}{ccc} \;b_{m}^{\mathrm{NLoS}}\; & = & \;\frac{\Omega_{TR}^{\mathrm{NLoS}}\left(t\right)}{2\Omega_{TR}^{\mathrm{RIS}}\left(t\right)+2\Omega_{TR}^{\mathrm{NLoS}}\left(t\right)}{\left(\frac{2\pi }{\lambda }\right)}^{m}\int_{-\pi /2}^{\pi /2}\int_{-\pi }^{\pi }f\left(\alpha_{R},\beta_{R}\right)[e^{j\varphi_{\ell }-j\frac{2\pi }{\lambda }\left(\xi_{T,\mathrm{cluster},\ell }\left(t\right)+\xi_{R,\mathrm{cluster},\ell }\left(t\right)\right)}\\ & & \;\times \;e^{j\frac{2\pi }{\lambda }\left(v_{T}\cos<v_{T},d_{T,\mathrm{cluster},\ell }\left(t\right)>+v_{R}\cos<d_{R,\mathrm{cluster},\ell }\left(t\right),v_{R}>\right)t}{]}^{m}d\alpha_{R}d\beta_{R}. \end{array}$$

For m=0, we immediately have

(30) $$\begin{array}{cc} b_{0}^{\mathrm{RIS}} & =\frac{\Omega_{TR}^{\mathrm{RIS}}\left(t\right)}{2\Omega_{TR}^{\mathrm{RIS}}\left(t\right)+2\Omega_{TR}^{\mathrm{NLoS}}\left(t\right)}, \end{array}$$

(31) $$\begin{array}{cc} b_{0}^{\mathrm{NLoS}} & =\frac{\Omega_{TR}^{\mathrm{NLoS}}\left(t\right)}{2\Omega_{TR}^{\mathrm{RIS}}\left(t\right)+2\Omega_{TR}^{\mathrm{NLoS}}\left(t\right)}, \end{array}$$

which leads to

(32) $$b_{0}=b_{0}^{\mathrm{RIS}}+b_{0}^{\mathrm{NLoS}}=\frac{1}{2}.$$

For m=1, using the formula for the first moment of the von Mises–Fisher (vMF) distribution [31], we can easily show that, for the RIS path, the spectral moment $b_{1}^{\mathrm{RIS}}$ can be specifically expressed as

(33) $$\begin{array}{cc} \;b_{1}^{\mathrm{RIS}}=b_{0}^{\mathrm{RIS}}\left(\frac{2\pi }{\lambda }\right)\int_{-\pi /2}^{\pi /2}\int_{-\pi }^{\pi }f\left(\alpha_{T},\beta_{T}\right)[e^{j\varphi_{m_{x},m_{z}}\left(t\right)-j\frac{2\pi }{\lambda }\left(\xi_{T,(m_{x},m_{z})}\left(t\right)+\xi_{R,(m_{x},m_{z})}\left(t\right)\right)}\\ & \;\times \;e^{j\frac{2\pi }{\lambda }\left(v_{T-\mathrm{RIS}}\left(t\right)\cos<v_{T-\mathrm{RIS}}\left(t\right),d_{T,m_{x},m_{z}}\left(t\right)>+v_{R-\mathrm{RIS}}\left(t\right)\cos<d_{R,m_{x},m_{z}}\left(t\right),v_{R-\mathrm{RIS}}\left(t\right)>\right)t}]d\alpha_{T}d\beta_{T}, \end{array}$$

and for the NLoS path, the spectral moment $b_{1}^{\mathrm{NLoS}}$ can be specifically expressed as

(34) $$\begin{array}{ccc} \;b_{1}^{\mathrm{NLoS}}\; & = & \;b_{0}^{\mathrm{NLoS}}\left(\frac{2\pi }{\lambda }\right)\int_{-\pi /2}^{\pi /2}\int_{-\pi }^{\pi }f\left(\alpha_{R},\beta_{R}\right)[e^{j\varphi_{\ell }-j\frac{2\pi }{\lambda }\left(\xi_{T,\mathrm{cluster},\ell }\left(t\right)+\xi_{R,\mathrm{cluster},\ell }\left(t\right)\right)}\\ & & \;\times \;e^{j\frac{2\pi }{\lambda }\left(v_{T}\left(t\right)\cos<v_{T}\left(t\right),d_{T,\mathrm{cluster},\ell }\left(t\right)>+v_{R}\left(t\right)\cos<d_{R,\mathrm{cluster},\ell }\left(t\right),v_{R}\left(t\right)>\right)t}]d\alpha_{R}d\beta_{R}. \end{array}$$

For m=2, using the formulas regarding the first moment and the second moment of the vMF distribution, we can obtain the final expressions of $b_{2}^{\mathrm{RIS}}$ as follows:

(35) $$\begin{array}{cc} \;b_{2}^{\mathrm{RIS}}=b_{0}^{\mathrm{RIS}}{\left(\frac{2\pi }{\lambda }\right)}^{2}\int_{-\pi /2}^{\pi /2}\int_{-\pi }^{\pi }f\left(\alpha_{T},\beta_{T}\right)[e^{j\varphi_{m_{x},m_{z}}\left(t\right)-j\frac{2\pi }{\lambda }\left(\xi_{T,(m_{x},m_{z})}\left(t\right)+\xi_{R,(m_{x},m_{z})}\left(t\right)\right)}\\ & \;\times \;e^{j\frac{2\pi }{\lambda }\left(v_{T-\mathrm{RIS}}\left(t\right)\cos<v_{T-\mathrm{RIS}}\left(t\right),d_{T,m_{x},m_{z}}\left(t\right)>+v_{R-\mathrm{RIS}}\left(t\right)\cos<d_{R,m_{x},m_{z}}\left(t\right),v_{R-\mathrm{RIS}}\left(t\right)>\right)t}{]}^{2}d\alpha_{T}d\beta_{T}, \end{array}$$

and for the NLoS path, the spectral moment $b_{2}^{\mathrm{NLoS}}$ can be specifically expressed as

(36) $$\begin{array}{ccc} \;b_{2}^{\mathrm{NLoS}}\; & = & \;b_{0}^{\mathrm{NLoS}}{\left(\frac{2\pi }{\lambda }\right)}^{2}\int_{-\pi /2}^{\pi /2}\int_{-\pi }^{\pi }f\left(\alpha_{R},\beta_{R}\right)[e^{j\varphi_{\ell }-j\frac{2\pi }{\lambda }\left(\xi_{T,\mathrm{cluster},\ell }\left(t\right)+\xi_{R,\mathrm{cluster},\ell }\left(t\right)\right)}\\ & & \;\times \;e^{j\frac{2\pi }{\lambda }\left(v_{T}\left(t\right)\cos<v_{T}\left(t\right),d_{T,\mathrm{cluster},\ell }\left(t\right)>+v_{R}\left(t\right)\cos<d_{R,\mathrm{cluster},\ell }\left(t\right),v_{R}\left(t\right)>\right)t}{]}^{2}d\alpha_{R}d\beta_{R}. \end{array}$$

**Figure 3:**
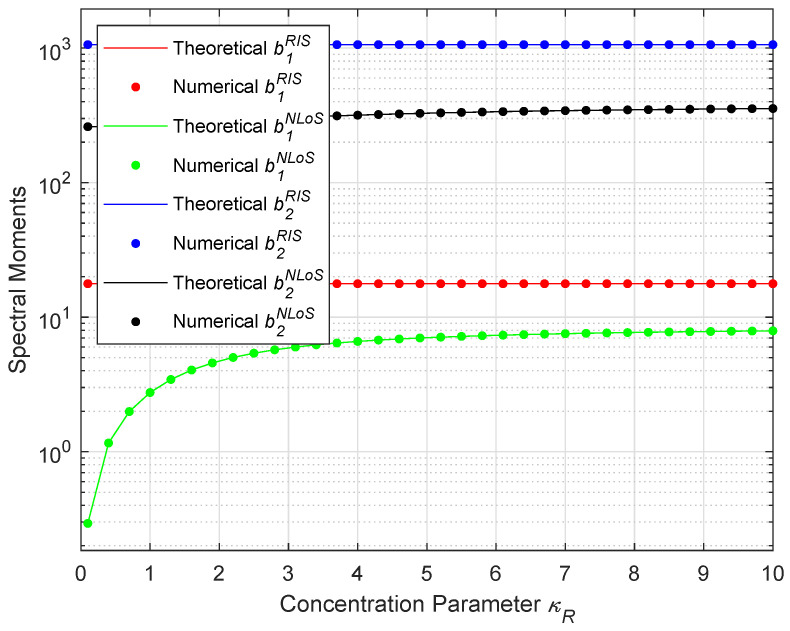
Comparison of the spectral moments between the theoretical expressions and the numerical results.

To demonstrate the correctness of the theoretical expressions for the spectral moments, we compare them with the numerical results. The comparison is shown in Figure 3, where $f_{c}=3\;\mathrm{GHz}$, $v_{T}={\left[\cos20^{\circ },\sin20^{\circ },0\right]}^{T}\;m/s$, $v_{R}={\left[\cos40^{\circ },\sin40^{\circ },0\right]}^{T}\;m/s$, $\kappa_{\mathrm{RIS}}=20$, $\alpha_{T\mu }=70^{\circ }$, $\alpha_{R\mu }=20^{\circ }$, $\beta_{T\mu }=\beta_{R\mu }=10^{\circ }$, $\eta_{\mathrm{RIS}}=0.6$, and K=0.01. We have defined $\alpha_{T\mu }$ and $\alpha_{R\mu }$ as the mean angles at which the scatterers are projected to the horizontal plane; $\beta_{T\mu }-\beta_{Tm}=\beta_{T\min}$ and $\beta_{T\mu }+\beta_{Tm}=\beta_{T\max}$, in which $\beta_{T\mu }$ and $\beta_{R\mu }$ denote mean elevation angles of scatterers; $\beta_{Tm}$ and $\beta_{Rm}$ denote spread parameters of elevation angles, respectively. It can be found that the excellent agreement validates the accuracy of the derivation of the spectral moments of the proposed channel model, which is also in line with the analytical findings in [30]. Hence, the correctness of the theoretical derivations is confirmed.

## 4. Simulated LCR of V2V Channels

In this section, we show the simulated LCR that correspond to the theoretical ones. The key to simulating the channel characteristics is to generate, under a finite number of scatterers, the discrete AoDs and AoAs $\alpha_{T}^{\left(n\right)}$, $\beta_{T}^{\left(n\right)}$, $\alpha_{R}^{\left(n\right)}$, and $\beta_{R}^{\left(n\right)}$. There are mainly two kinds of simulation models to do this: the deterministic one and the stochastic one. In the deterministic simulation model, the discrete AoDs and AoAs are generated as follows:

(37) $$\begin{array}{cc} \alpha_{T}^{\left(n\right)} & =F_{\alpha_{T}}^{-1}\left(\frac{n-\frac{1}{2}}{N}\right), \end{array}$$

(38) $$\begin{array}{cc} \alpha_{R}^{\left(n\right)} & =F_{\alpha_{R}}^{-1}\left(\frac{n-\frac{1}{2}}{N}\right), \end{array}$$

(39) $$\begin{array}{cc} \beta_{T}^{\left(n\right)} & =\beta_{T\mu }+\frac{2\beta_{Tm}}{\pi }\cdot \arcsin\left(\frac{2(n-\frac{1}{2})}{N}-1\right), \end{array}$$

(40) $$\begin{array}{cc} \beta_{R}^{\left(n\right)} & =\beta_{R\mu }+\frac{2\beta_{Rm}}{\pi }\cdot \arcsin\left(\frac{2(n-\frac{1}{2})}{N}-1\right), \end{array}$$

for n=1,2,…,N. Here, $F_{\alpha_{T}}^{-1}\left(\;\cdot \;\right)$ and $F_{\alpha_{R}}^{-1}\left(\;\cdot \;\right)$ denote the inverse functions of the von Mises cumulative distribution functions for $\alpha_{T}^{\left(n\right)}$ and $\alpha_{R}^{\left(n\right)}$, respectively.

The simulated LCR can be expressed as

(41) $$\begin{array}{cc} \; & \;\hat{L}\left(r\right)=\frac{2r\sqrt{K+1}}{\pi^{\frac{3}{2}}}\sqrt{\frac{{\hat{b}}_{2}}{{\hat{b}}_{0}}-\frac{{\hat{b}}_{1}^{2}}{{\hat{b}}_{0}^{2}}}e^{-K-\left(K+1\right)r^{2}}\\ \; & \times \int_{0}^{\frac{\pi }{2}}\cosh\left(2\sqrt{K\left(K+1\right)}r\cos\theta \right)\left[e^{-{\left(\hat{\chi }\sin\theta \right)}^{2}}+\sqrt{\pi }\hat{\chi }\sin\theta \cdot \mathrm{erf}\left(\hat{\chi }\sin\theta \right)\right]d\theta , \end{array}$$

in which $\hat{\chi }$ is given by $\hat{\chi }=\sqrt{\frac{K{\hat{b}}_{1}^{2}}{{\hat{b}}_{0}{\hat{b}}_{2}-{\hat{b}}_{1}^{2}}}$ and ${\hat{b}}_{m}$ (m=0,1,2) are given as

(42) $${\hat{b}}_{m}\;=\;{\hat{b}}_{\mathrm{RIS},\;m}\;+\;{\hat{b}}_{\mathrm{NLoS},\;m},\;$$

where, for the RIS path,

(43) $$\begin{array}{cc} \;{\hat{b}}_{\mathrm{RIS},m}=\frac{\eta_{\mathrm{RIS}}}{2(K+1)}{\left(\frac{2\pi }{\lambda }\right)}^{m}\frac{1}{M_{x}M_{z}}\sum_{m_{x}=1}^{M_{x}}\sum_{m_{z}=1}^{M_{z}}[e^{j\varphi_{m_{x},m_{z}}\left(t\right)-j\frac{2\pi }{\lambda }\left(\xi_{T,(m_{x},m_{z})}\left(t\right)+\xi_{R,(m_{x},m_{z})}\left(t\right)\right)}\\ & \;\times \;e^{j\frac{2\pi }{\lambda }\left(v_{T-\mathrm{RIS}}\left(t\right)\cos<v_{T-\mathrm{RIS}}\left(t\right),d_{T,m_{x},m_{z}}\left(t\right)>+v_{R-\mathrm{RIS}}\left(t\right)\cos<v_{R,m_{x},m_{z}}\left(t\right),d_{R-\mathrm{RIS}}\left(t\right)>\right)t}{]}^{m}, \end{array}$$

and for the NLoS path, there is

(44) $$\begin{array}{ccc} \;{\hat{b}}_{\mathrm{NLoS},m}\; & = & \;\frac{\eta_{\mathrm{NLoS}}}{2(K+1)}{\left(\frac{2\pi }{\lambda }\right)}^{m}\frac{1}{L}\sum_{\ell =1}^{L}[e^{j\varphi_{\ell }-j\frac{2\pi }{\lambda }\left(\xi_{T,\mathrm{cluster},\ell }\left(t\right)+\xi_{R,\mathrm{cluster},\ell }\left(t\right)\right)}\\ & & \;\times \;e^{j\frac{2\pi }{\lambda }\left(v_{T}\left(t\right)\cos<v_{T}\left(t\right),d_{T,\mathrm{cluster},\ell }\left(t\right)>+v_{R}\left(t\right)\cos<d_{R,\mathrm{cluster},\ell }\left(t\right),v_{R}\left(t\right)>\right)t}{]}^{m}. \end{array}$$

In the meantime, the simulated AFD can be written as

(45) $$\hat{T}\left(r\right)=\frac{1-Q\left(\sqrt{2K},\sqrt{2\left(K+1\right)r^{2}}\right)}{\hat{L}\left(r\right)},$$

which can be easily obtained by substituting the simulated LCR given by (41).

## 5. Analysis and Discussions

In this section, we further provide theoretical insights into the impact of RIS motion direction and scatterer distribution on the level crossing rate characteristics, in addition to the numerical simulations. Recall from (25) that the LCR is determined by the spectral moments $b_{0},b_{1}$, and $b_{2}$, which are composed of the RIS-assisted and NLoS scattering contributions as given in (27)–(36). In particular, the dominant factor affecting the envelope dynamics is the coefficient

(46) $$\begin{array}{c} \sqrt{\frac{b_{2}}{b_{0}}-\frac{b_{1}^{2}}{b_{0}^{2}}}. \end{array}$$

For simplicity, we consider two typical cases: (1) the RIS reflection-dominated case, where $\Omega_{\mathrm{RIS}}\gg \Omega_{\mathrm{NLoS}}$; (2) the NLoS scattering-dominated case, where $\Omega_{\mathrm{NLoS}}\gg \Omega_{\mathrm{RIS}}$.

### 5.1. RIS-Dominated Case

In the RIS-dominated case, the spectral moments are mainly contributed by $b_{m}^{\mathrm{RIS}}$. Following (33) and (35), the expression $b_{0}^{\mathrm{RIS}}b_{2}^{\mathrm{RIS}}-{\left(b_{1}^{\mathrm{RIS}}\right)}^{2}$ can be expanded as

(47) $$\begin{array}{c} b_{0}^{\mathrm{RIS}}b_{2}^{\mathrm{RIS}}-{\left(b_{1}^{\mathrm{RIS}}\right)}^{2}={\left(b_{0}^{\mathrm{RIS}}\right)}^{2}{\left(\frac{2\pi }{\lambda }\right)}^{2}\left[\Gamma_{\mathrm{RIS}}\left(v_{\mathrm{RIS}},\alpha_{T},\beta_{T},\alpha_{R},\beta_{R}\right)\right], \end{array}$$

where $\Gamma_{\mathrm{RIS}}\left(\cdot \right)$ denotes a quadratic form that depends on the projections of the RIS velocity vector onto the dominant departure and arrival angles. It can be shown that the LCR achieves its minimum when these velocity projections are maximized, i.e., when the RIS moves directly along or opposite to the main propagation direction:

(48) $$\left\{\begin{array}{c} \gamma_{\mathrm{RIS}}-\alpha_{R\mu }=0^{\circ },\\ \xi_{\mathrm{RIS}}-\beta_{R\mu }=0^{\circ }, \end{array}\right.\;\mathrm{or}\;\left\{\begin{array}{c} \gamma_{\mathrm{RIS}}-\alpha_{R\mu }=180^{\circ },\\ \xi_{\mathrm{RIS}}+\beta_{R\mu }=0^{\circ }. \end{array}\right.$$

### 5.2. NLoS-Dominated Case

In the NLoS-dominated case, $b_{m}^{\mathrm{NLoS}}$ becomes dominant. The same analysis leads to

(49) $$\begin{array}{c} b_{0}^{\mathrm{NLoS}}b_{2}^{\mathrm{NLoS}}-{\left(b_{1}^{\mathrm{NLoS}}\right)}^{2}={\left(b_{0}^{\mathrm{NLoS}}\right)}^{2}{\left(\frac{2\pi }{\lambda }\right)}^{2}\left[\Gamma_{\mathrm{NLoS}}\left(v_{T},v_{R},\alpha_{T},\beta_{T},\alpha_{R},\beta_{R}\right)\right]. \end{array}$$

It follows that the lowest LCR occurs when the Tx/Rx velocity vector is aligned with the dominant scattering clusters, i.e.,

(50) $$\left\{\begin{array}{c} \gamma_{T}-\alpha_{T\mu }=0^{\circ },\\ \xi_{T}-\beta_{T\mu }=0^{\circ }, \end{array}\right.\;\mathrm{or}\;\left\{\begin{array}{c} \gamma_{R}-\alpha_{R\mu }=0^{\circ },\\ \xi_{R}-\beta_{R\mu }=0^{\circ }. \end{array}\right.$$

From the above analysis, it is concluded that the motion direction of RIS (or UAV) plays a critical role in shaping the Doppler spread, and hence the LCR. Specifically, aligning the motion with the dominant AoA/AoD directions results in a broader Doppler distribution and a higher LCR profile, whereas orthogonal motion leads to a narrower LCR curve. These theoretical results are consistent with the simulated LCR curves shown below.

### 5.3. Numerical Results and Discussions

**Figure 4:**
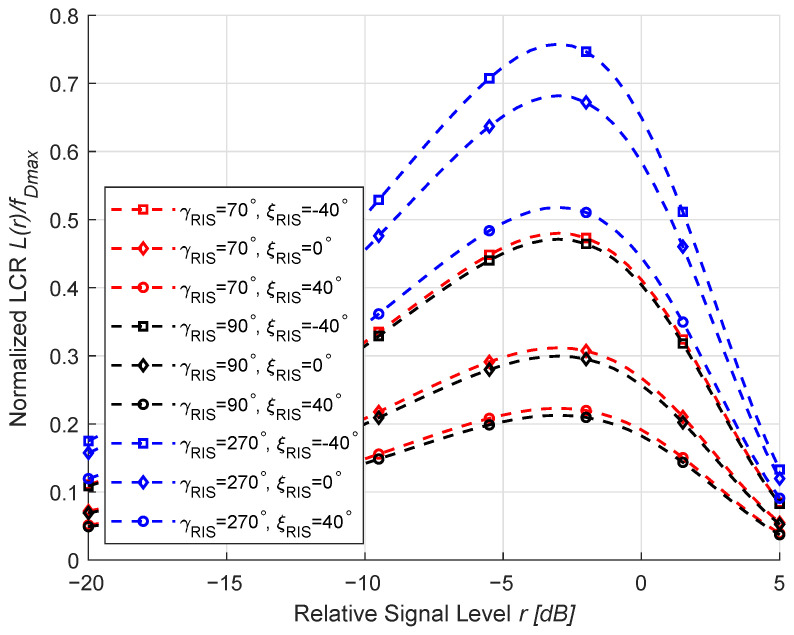
LCRs for different moving directions of the RIS under *K* = 0.01, $\eta_{\mathrm{RIS}}$ = 0.9, and $\eta_{\mathrm{NLoS}}$ = 0.1.

We exhibit in Figure 4 the LCRs for different moving directions of the RIS under a weak LoS environment with rich scatterers when K=0.01, $M_{x}=M_{z}=10$, $\eta_{\mathrm{RIS}}=0.9$, and $\eta_{\mathrm{NLoS}}=0.1$. It is stated in [3] that the vehicle speed ranges from a few meters to a few hundred meters per second; accordingly, we investigate the LCRs of the proposed channel model under different speeds in Figure 4. It can be observed that when the MT and MR are relatively static, the LCR curves vary considerably with the RIS motion direction. This occurs because altering the RIS motion direction changes the projection of its velocity vector onto the propagation paths, thus modifying the Doppler frequency distribution. When the motion direction aligns more closely with the dominant propagation path, the Doppler spread increases, leading to a higher and broader LCR profile. Conversely, when the motion direction is nearly orthogonal to the main path, the Doppler spread decreases, resulting in a narrower LCR curve. These findings align with the simulation results in [32], further validating the theoretical derivations.

**Figure 5:**
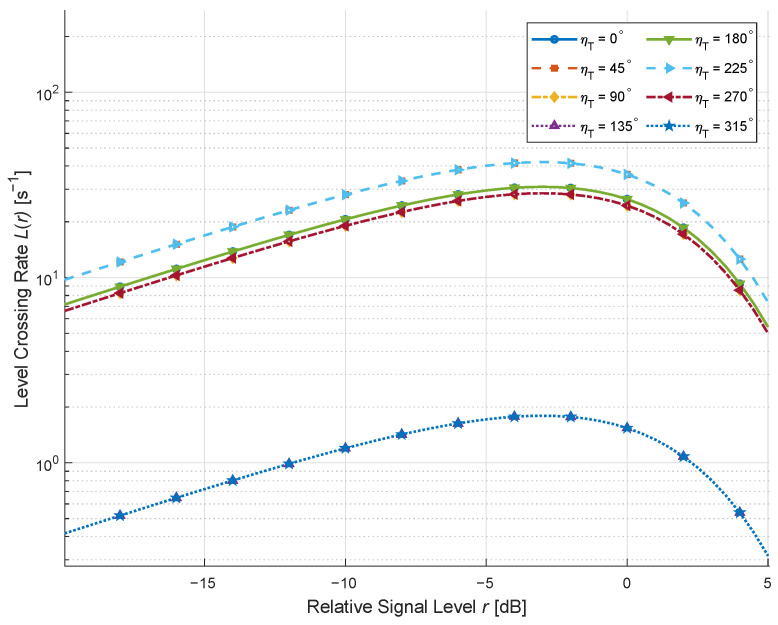
LCRs for different moving directions of the MT under *K* = 0.01, $\eta_{\mathrm{RIS}}$ = 0.9, and $\eta_{\mathrm{NLoS}}$ = 0.1.

We exhibit in Figure 5 the LCRs for different moving directions of the MT under a weak LoS environment with rich scatterers. The other parameters are $f_{c}=3$ GHz and $v_{T}=1$ m/s. Similar to the conclusion of [19], when the MT moves more closely along the dominant propagation direction, the Doppler spread is enlarged, yielding a higher and broader LCR profile. In contrast, when the MT motion is nearly orthogonal to the main path, the Doppler spread becomes smaller, and the LCR curves shrink accordingly. These results further validate the theoretical derivations.

**Figure 6:**
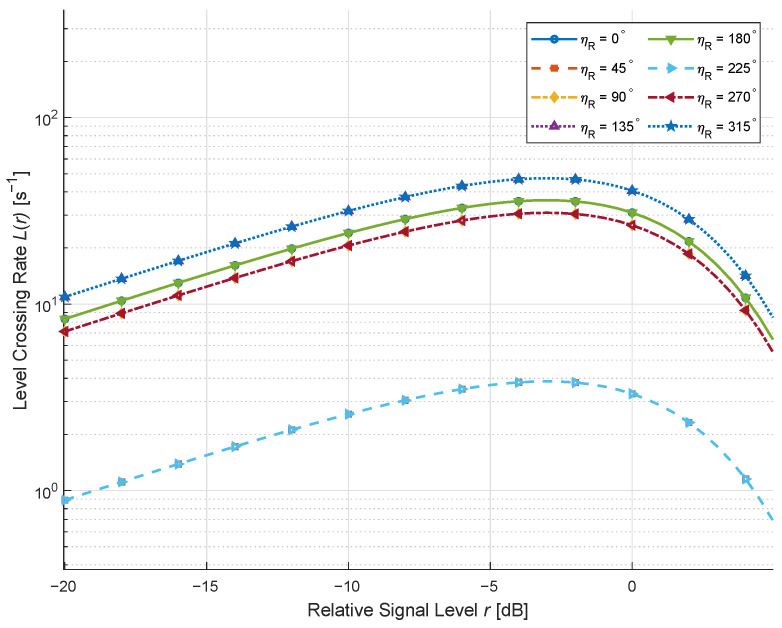
LCRs for different moving directions of the MR under *K* = 0.01, $\eta_{\mathrm{RIS}}$ = 0.9, and $\eta_{\mathrm{NLoS}}$ = 0.1.

We exhibit in Figure 6 the LCRs for different moving directions of the MT under a weak LoS environment with rich scatterers. Similar to the MT case, the projection of the MR velocity onto the propagation paths governs the Doppler frequency spread. When the MR motion direction aligns with the main arrival direction, the Doppler dispersion is maximized, leading to a more rapid fluctuation of the envelope and hence a larger LCR. On the other hand, orthogonal motion directions yield reduced Doppler spread and narrower LCR curves, which is identical to [19,33], indicating the correctness of our results.

**Figure 7:**
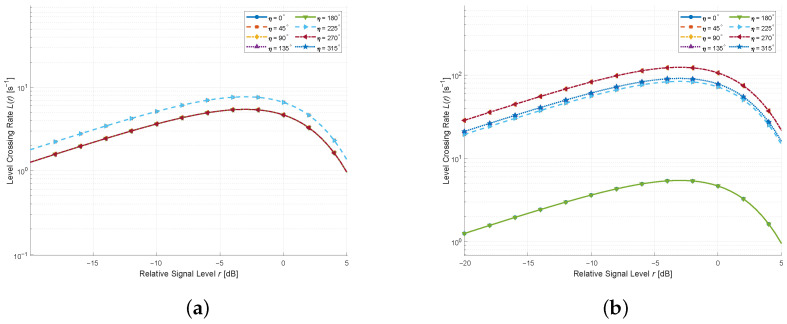
LCR for MT/MR and RIS moving in different directions (K=0.01): (**a**) Same direction; (**b**) Opposite direction.

We present in Figure 7 the LCRs for different moving directions of the MT/MR and RIS under K=0.01. The other parameters used for Figure 7 are $f_{c}=3$ GHz, $v_{T}=1$ m/s, $v_{R}=1$ m/s, and $v_{\mathrm{RIS}}=1$ m/s. It can be seen that when both the MT/MR and RIS move in the same direction, the Doppler components add coherently, producing more pronounced LCR variations. Conversely, when the MT/MR and RIS move in opposite directions, their Doppler contributions may partially counteract, leading to relatively reduced LCR levels. These results highlight the coupled impact of multiple moving entities on the second-order statistics, which agrees with [27,28], further verifying the correctness of the theoretical derivations.

**Figure 8:**
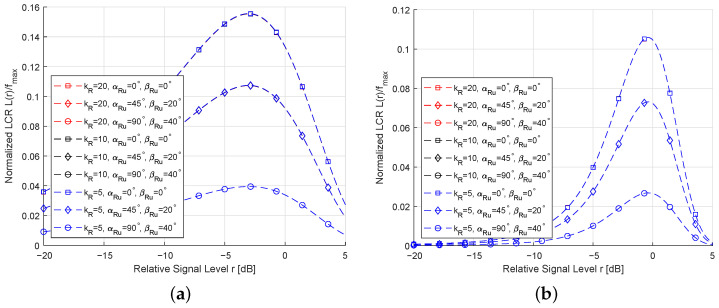
LCRs for different values of the vMF concentration parameter: (**a**) K=0.01; (**b**) K=5.

We present in Figure 8 the LCRs for different values of $\kappa_{\mathrm{RIS}}$, $\alpha_{R\mu }$, and $\beta_{R\mu }$ under K=0.01 (weak LoS) or K=5 (strong LoS). As shown in Figure 8, the LCR changes noticeably with variations in the vMF parameters, indicating that the scatterer distribution plays a crucial role in the temporal behavior of the channel. Specifically, increasing $\kappa_{\mathrm{RIS}}$ makes the scatterer distribution more concentrated around the mean direction, leading to a steeper LCR curve. Comparing Figure 8a,b, it can be observed that as *K* increases, the impact of $\kappa_{\mathrm{RIS}}$ on the LCR becomes weaker, because the LoS component dominates the received signal and reduces the relative influence of the scattered components on the second-order statistics. These findings are consistent with the computer simulation results in [34,35], thus verifying the accuracy of the derived expressions.

## 6. Conclusions

In this paper, we have developed a comprehensive 3D channel model tailored for RIS-assisted V2V communication systems, which explicitly incorporates the mobility of the transmitter, receiver, and UAV-mounted RIS. Numerical evaluations verify that the proposed model effectively captures time-varying propagation behaviors, as well as the influence of RIS motion direction and scatterer distribution on the LCR. The model offers accurate analytical expressions for the LCR under Rician fading conditions and provides computationally efficient deterministic and stochastic simulation frameworks for practical deployment. Moreover, our analysis reveals that motion alignment of the RIS and the spatial distribution of scatterers substantially affect Doppler spread and fading dynamics, highlighting the necessity of optimized RIS configuration and mobility management in realistic V2V environments. Collectively, these findings confirm that RIS deployment can significantly enhance the performance of RIS-assisted V2V systems, thereby offering actionable insights for the design and performance assessment of such communication architectures.

Building upon the proposed channel model, future work will focus on leveraging this framework to investigate achievable performance gains in energy efficiency through the joint optimization of RIS phase shifts and UAV trajectory. Such theoretical and simulation-based studies are expected to further inform practical deployment strategies for dynamic V2V networks.

## Figures and Tables

**Table 1:** Summary and comparison of representative for beam domain channel modeling.

Ref.	Summary
[13]	Proposed a 3D non-stationary channel model for RIS-empowered MIMO V2V communication scenarios by utilizing beamforming operations based on the discrete Fourier transform to transform the model from the array domain to the beam domain, which aims to reduce the computational complexity of RIS-empowered V2V channels.
[14]	Presented a comparative performance assessment of these channel modeling techniques in the context of RIS-assisted V2V communications at mmWave frequency, which aims to evaluate the ability of RT and GBSM to accurately capture the key features of RIS-assisted channels in an urban vehicular environment.
[15]	Presented a comparative performance assessment of channel modeling techniques in the context of RIS-assisted V2V communications at mmWave frequency, which aimed to evaluate the ability of RT and GBSM to accurately capture the key features of RIS-assisted channels in an urban vehicular environment.
[16]	Proposed a RIS-assisted NOMA network for the vehicular applications to harvest the spatial diversity of the RIS for improving the channel condition.
[17]	(a) Provided the marginal distributions of the signal-to-noise ratio and signal-to-interference-plus-noise ratio at the legitimate receiver and eavesdropper vehicles with the help of the CLT; (b) Derived the asymptotic expressions of the ASC in the high-SNR regime to acquire deeper understanding of the performance of the obtained secrecy metrics.
This paper	(a) Incorporates both the controllable RIS reflection link and the scattered NLoS link from clusters, providing a holistic view of the propagation environment; (b) Derives the time-varying expressions for crucial parameters, including propagation distances, AoDs, AoAs, and complex impulse responses for both the RIS and cluster links, capturing the inherent temporal non-stationarity; (c) Derives exact analytical expressions for the key second-order statistic, the LCR of the envelope for the proposed Rician fading channel model; (d) Investigates the impact of various system parameters, such as the motion direction of the RIS and the distribution of scatterers on the LCR.

## Data Availability

The original contributions presented in this study are included in the article. Further inquiries can be directed to the corresponding author.
